# Autoantibodies to muscarinic acetylcholine receptors found in patients with primary biliary cirrhosis

**DOI:** 10.1186/1471-230X-10-120

**Published:** 2010-10-16

**Authors:** Christoph P Berg, Karin Blume, Kirsten Lauber, Michael Gregor, Peter A Berg, Sebastian Wesselborg, Gerburg M Stein

**Affiliations:** 1 Department of Internal Medicine I, Medical Clinic, University of Tübingen, Germany

## Abstract

**Background:**

Autoantibodies to the human muscarinic acetylcholine receptor of the M3 type (hmAchR M3) have been suggested to play an etiopathogenic role in Sjögren's syndrome. Primary biliary cirrhosis (PBC) often is associated with this syndrome. Therefore, we studied the co-presence of hmAchR M3 autoantibodies in patients with PBC.

**Methods:**

Frequency of hmAchR M3 autoantibodies was assessed by Western blotting analysis as well as by an ELISA using a 25-mer peptide of the 2^nd ^extracellular loop of hmAchR M3. Co-localization of hmAchR M3/PBC-specific autoantibodies was studied by confocal laser scanning microscopy. Finally, sera from patients with PBC as well as from healthy controls were tested.

**Results:**

Western blotting analysis as well as results from ELISA testing revealed a significantly enhanced IgG reactivity in PBC patients in contrast to healthy controls. Co-localization of autoantibodies with the hmAchR M3 receptor-specific autoantibodies was observed in 10 out of 12 PBC-patients but none of the 5 healthy controls. Antibodies of the IgM type were not found to be affected.

**Conclusions:**

For the first time, our data demonstrate the presence of autoantibodies to the hmAchR M3 in PBC patients. These findings might contribute to the understanding of the pathogenesis of this disease. Further studies have to focus on the functionality of hmAchR M3 autoantibodies in PBC patients.

## Background

Primary biliary cirrhosis (PBC) is an autoimmune liver disease characterized by chronic progressive destruction of the small intrahepatic bile ducts [1-4]. Its etiopathogenesis still remains unclear, although (i) genetic disposition, (ii) microorganisms, (iii) apoptotic processes, as well as (iv) environmental factors have been suggested to be of relevance for both development and maintenance of PBC [2,5-10].

Diagnostically, antimitochondrial antibodies (AMA) which mainly target the different subunits of the pyruvate dehydrogenase complex (PDC) play an important role and have been shown to occur in about 90% of all PBC patients [1-3,11]. However, these antibodies do not meet the classical criteria for an autoantibody-mediated autoimmune disease [12-15], i.e., induction of the disease in animal models by passive transfer of the disease-specific antibodies or *via *the application of the target antigen and the recovery from the disease due to a reduction of the titers of the disease-specific antibodies [3,16-19]. Therefore, the PDC-specific antibodies seem to be of no etiopathogenic relevance. Furthermore, since PDC is an antigen expressed in almost all cell types they do not explain the organ-specificity of PBC.

Deduced from recent studies on other autoimmune disorders, a novel etiopathogenic concept has been developed which is based on the involvement of functionally active autoantibodies against neurotransmitter receptors [20]. As an example, patients with *Pemphigus vulgaris *exhibit autoantibodies to the alpha-9-acetylcholine-receptor which are responsible for the typical acantholysis [21]. In addition, experimental and clinical studies verify the pathogenic role of antibodies to the beta_1_-adrenergic receptor in dilatative cardiomyopathy [22]. Furthermore, in patients suffering from *myasthenia gravis*, autoantibodies to the alpha-1 subunit of the nicotinic acetylcholine receptor in muscles were shown to disturb neuromuscular signal transduction and mark the cells for complement mediated lysis [23]. Interestingly, also in patients with *M. Sjögren*, an autoimmune disease quite often being associated with PBC [24,25], autoantibodies to human muscarinic acetylcholine receptors (hmAchR) of the M3 type were suggested to be one factor responsible for disease induction [26,27]. Moreover, since this specific receptor subtype was also detected on biliary cells but not on hepatocytes [28,29] we hypothesized that hmAchR M3-specific autoantibodies could play an important role in the etiopathogenesis of PBC. Thus, we now have undertaken a comprehensive study analyzing whether autoantibodies to the hmAchR of the M3 type could also be found in patients with PBC.

## Methods

### Patients

Our well-characterized PBC cohort at University Hospital Tübingen encompasses 50 patients (42 female, 8 male); furthermore, also 16 healthy controls gave their informed consent for this study, which was approved by the local ethics committee.

PBC patients: mean age was 57.7 ± 10.8 years (range 27 - 74 years); all patients exhibited typical PBC-associated laboratory parameters (such as elevated levels of alkaline phosphatase (AP), γ-glutamyltransferase (gGT), and/or IgM values). Liver biopsies had been performed in 23 patients and demonstrated PBC-specific lesions in all instances. 48 patients showed a positive reaction in the immunofluorescence test (IFT) to mitochondrial antigens on cryostat sections (AMA-positivity); in the remaining 2 AMA-negative patients PBC was evidenced either by liver biopsy or the presence of anti-PDC-antibodies by Western blotting analysis. 20 patients showed ANA (anti-nuclear antigen) reactivity in the IFT. 13 patients exhibited SMA (smooth muscle antigen) reactivity in the IFT. Elevation of IgM globulins were observed in 37 patients (> 230 mg/dl) and elevation of IgG levels in 14 patients (> 1.600 mg/dl). 44 patients were under therapy with ursodeoxycholic acid.

Controls: sera from 16 healthy blood donors from the University Hospital Tübingen were included in our study (female-to-male ratio was 10:6; mean age: 32 ± 8 years; range 20 - 48 years). All sera were checked for autoantibody reactivity by the IFT and were found to be serologically negative for AMA. 3 patients showed autoantibodies to ANA and another 3 patients to SMA.

### Confocal Laser Scanning Microscopy

Co-localization of PBC-specific antibodies with autoantibodies to the hmAchR M3 was studied using a Leica TCS SP2-x1 confocal laser scanning microscope and 'Leica confocal' software (Leica Biosystems GmbH, Nussloch, Germany). For this purpose, HT-29 human colon carcinoma cells were cultured in RPMI-1640 medium (BioWhittaker, Verviers, Belgium) supplemented with 10% heat-inactivated fetal calf serum (PAA Laboratories, Cölbe, Germany), 100 units of penicillin/ml, 0.1 mg streptomycin/ml and 10 mM HEPES (all from Gibco, Karlsruhe, Germany). Cells were grown at 37 °C in a 5% CO_2 _atmosphere and maintained in the log phase. Using a polyclonal anti-hmAchR M3 antibody, HT-29 cells were tested by Western blot analyses for expression of hmAchR of the M3 type (data not shown). They were seeded at 5 × 10^5^/ml in 24 wells plates containing a sterile cover slip. Cells were allowed to adhere overnight and then washed twice with PBS (Gibco). Cells were fixed with 500 μl of 3.7% paraformaldehyde (Sigma, Deisenhofen, Germany) for 20 min at room temperature (RT) followed by three washing steps with 1 ml PBS/0.5% BSA (P-BSA), and blocking for 10 min in PBC/10% FCS at RT. After washing once with P-BSA, cells were permeabilized with 0.1% Triton X-100 (Roth, Karlsruhe, Germany) for 10 min at RT, washed three times with P-BSA. Labelling with primary antibodies (50 μl; 60 min at RT) employed (i) a rabbit anti-hmAchR M3 polyclonal antibody (poAb; Acris, Hiddenhausen, Germany), (ii) a mouse anti-Na^+^/K^+^-ATPase mAb (Biomol, Hamburg, Germany) and serum IgG fractions from (iii) PBC patients or (iv) healthy controls. Serum IgG from the respective individuals was purified as described [30] and diluted in PBS/1% BSA to a final concentration (f.c.) of 2.5 μg/ml.

After washing with P-BSA for three times, 50 μl of the secondary antibodies (goat anti-rabbit IgG, Alexa Fluor 488 conjugated (Invitrogen, Karlsruhe, Germany); goat anti-mouse IgG, Cy3 conjugated (Amersham Pharmacia, Freiburg, Germany); goat anti-human IgG, Alexa 633 conjugated (Invitrogen)) were added at a f.c. of 2 μg/ml in P-BSA for 30 min at RT in the dark. After three washing steps, cells were mounted on a slide (Langenbrinck, Emmendingen, Germany) using 7 μl mounting medium (Confocal-Matrix, Micro Tech Lab, Graz, Austria).

### Enzyme linked immunosorbent assay (ELISA)

Sera were tested employing a 25-mer peptide ([31-33]; synthesized by Seqlab, Göttingen, Germany; amino acids 213 - 237, KRTVPPGECFIQFLSEPTITFGTAI; concentration of 10 μg/ml) of the hmAchR M3 protein by ELISA according to the method described [34,35]. Sera were diluted 1:100 [36,37]. The secondary anti-human IgG or IgM antibodies (Dianova, Hamburg, Germany) were used at a concentration of 0.5 μg/ml. The cut-off values were defined as the mean of the values from the 16 controls in addition to 2 standard deviations.

### Western blot analysis

Sera were analyzed for the presence of autoantibodies to the hmAchR of the M3 type using membrane fractions of hmAchR M3 expressing Sf9 cells (Sigma, Deisenhofen, Germany) by Western blotting analysis following the method described [30,38]. Sera were diluted 1:200 in 5% milk powder (low fat; Roth, Karlsruhe, Germany) in TBST (10 mM Tris-HCl pH 7.4, 100 mM NaCl, 0.01% Triton). As secondary antibody horse radish peroxidase-conjugated anti-human IgG (1:4.000; DAKO, Hamburg, Germany) was used. Proteins were detected by the enhanced chemoluminescence (ECL) method (Amersham-Buchler, Germany).

### Statistics

Differences between the sera of the patient or control cohorts concerning reactivity to (i) the hmAchR of the M3 type or (ii) the 25-mer peptide were statistically evaluated using the Wilcoxon test (WinSTAT software); p ≤ 0.05 was defined as significant.

## Results

### Co-localization of the PBC-specific autoantibodies with autoantibodies to the hmAchR M3

**Figure 1:**
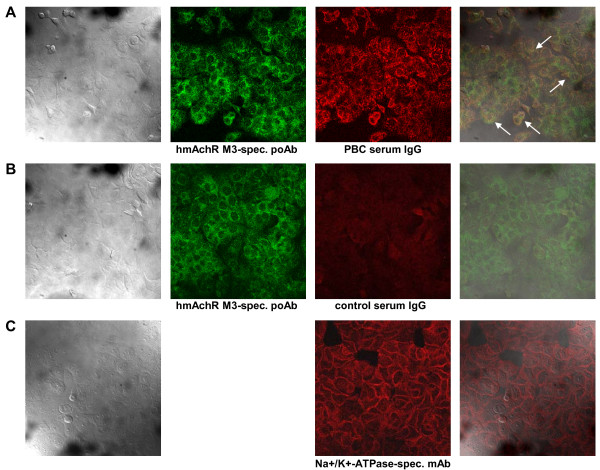
**Co-localization of hmAchR M3-specific antibodies with patient-derived PBC-specific antibodies**. Confocal laser scanning microscopy detecting co-localization of a hmAchR M3-specific antibody with patient-derived PBC-specific antibodies. HmAchR M3-positive HT-29 human colon carcinoma cells were fixed and stained with a hmAchR M3-specific polyclonal antibody (poAb) (lanes A+B, column 2) or the serum IgG fractions of a representative PBC patient (lane A, column 3) or a healthy control (lane B, column 3). As a positive control for cell surface receptor staining, also a Na^+^/K^+^-ATPase-specific mAb (lane C, column 3) was used. Magnification: 630×. Provided are the transmission (column 1), fluorescence data (columns 2+3) as well as the overlay (column 4) of the different photographs. The white arrows show the co-localization of both antibodies as indicated by the yellow color.

In order to investigate the presence of human muscarinic acetylcholine receptor M3 (hmAchR M3)-specific autoantibodies in PBC patients, we first addressed the question whether PBC-specific antibodies do co-localize with antibodies to the hmAchR M3. Therefore, HT-29 human colon carcinoma cells expressing the hmAchR M3 receptor (proven by Western blotting; data not shown) were fixed on cover slides and stained with sera from PBC patients (representative example given in Figure 1, lane A), or control sera IgG fractions from healthy donors (Figure 1, lane B) as well as a commercially available polyclonal antibody to the hmAchR M3 (Figure 1, lanes A+B, column 2); as a control for cell surface receptor staining, a Na^+^/K^+^-ATPase-specific antibody was used (Figure 1, lane C, column 3).

As a result, overlaying of the hmAchR M3-specific antibody staining with staining by sera from PBC patients (Figure 1, lane A, column 4) was found to exhibit a profound co-localization of detected antigens. In clear contrast, no co-localizing signals were found for the serum IgG fractions of healthy donors (Figure 1, lane B, column 4). In total, co-localization of PBC-specific antibodies with the hmAchR M3-specific antibody was found to be present in 10 out of 12 tested PBC sera, but none of the tested control sera (Tab. 1).

**Table 1:** Summary of the results of the confocal laser scanning microscopy*

	tested (n)	positive overlays (n)
PBC patients	12	10
healthy controls	5	0

* Co-localization of autoantibodies present in the serum IgG fractions of PBC patients and healthy controls being detected with a polyclonal antibody to the hmAchR M3 (studied on hmAchR M3-positive HT-29 human colon carcinoma cells).

In contrast to the staining with the Na^+^/K^+^-ATPase-specific mAb (Figure 1, lane C, column 3), staining by the polyclonal hmAchR M3 antibody was not restricted to the cell membrane; an additional diffuse intracellular staining was detectable (Figure 1, lanes A+B, column 2).

### Presence of autoantibodies to the hmAchR of the M3 type in sera of PBC patients

**Figure 2:**
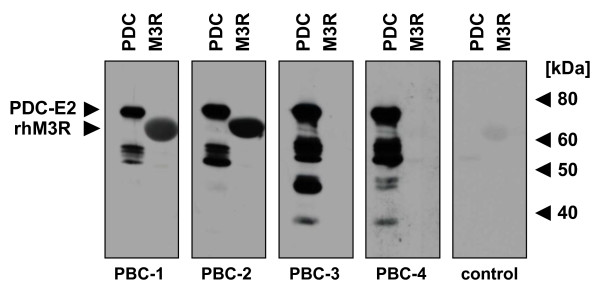
**Detection of hmAchR M3 autoantibodies in sera of PBC patients by Western Blot analysis**. Detection of autoantibodies to the recombinant human muscarinic acetylcholine receptor M3 (M3R) in the sera from PBC patients was revealed by Western blot analysis. M3R was derived from the membrane fraction of M3R over-expressing Sf9 cells (0.5 μg/lane). PDC (1 μg/lane) was used as control for the PBC sera. Sera were diluted 1:200 and visualized by the anti-human IgG secondary antibody. Given are exemplary the blots of two sera with positive (PBC-1 and 2) and two sera with negative results (PBC-3 and 4) to the M3R, as well as one negative control.

To assess whether PBC patients produce autoantibodies to the hmAchR of the M3 type, we next performed a Western blotting analysis using the membrane fraction of Sf9 cells over-expressing the recombinant hmAchR receptor protein (rhM3R) of the M3 type (Figure 2). While all sera from four initially analyzed PBC patients clearly detected the typical bands of the PDC complex (Figure 2, left lanes), hmAchR M3 receptor protein was recognized at 65 kDa by only 2 of the 4 PBC sera (Figure 2, right lanes). Employment of a control serum from a healthy donor did not show any prominent band (Figure 2, right panel).

Testing a larger cohort of PBC patients, we found a significantly elevated frequency of IgG type-autoantibodies to the recombinant hmAchR M3 receptor protein in sera from PDC-E2^+ ^PBC patients 33%) versus the control group comprising healthy donors (7.7%) (Tab. 2). As an exception, only one healthy control displayed a hmAchR M3-specific band, while, as expected, none of the different subunits of the PDC was recognized by the same serum (data not shown).

**Table 2:** Frequency of IgG type-autoantibodies to the recombinant hmAchR M3 receptor protein in sera from PDC-E2^+ ^PBC patients and healthy controls*

	Tested (n)	positive (n)	positive (%)
PBC patients	27	9	33**
healthy controls	13	1	7.7

* analyses were performed by Western blotting using the membrane fraction of hmAchR M3 over-expressing Sf9 cells;

** significantly different from the controls (p = 0.043; Wilcoxon test).

### Frequency of hmAchR M3-specific autoantibodies in the sera of patients with PBC

**Figure 3:**
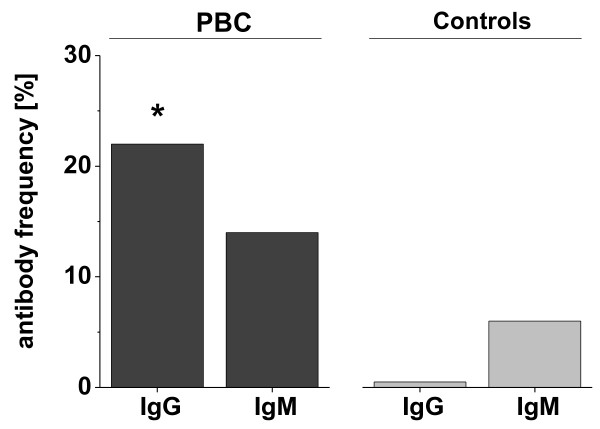
**Detection of hmAchR M3 autoantibodies in sera of PBC patients by ELISA**. Frequency of hmAchR M3-specific autoantibodies in sera of PBC patients (n = 50) and healthy controls (n = 16) were detected by ELISA: antibodies of the IgG- and IgM-type to the 25-mer peptide of the 2nd extracellular loop of the hmAchR M3. *p < 0.05 as compared to the healthy controls (Wilcoxon test).

The frequency of autoantibodies to hmAchR M3 receptor was also assessed by an ELISA using a 25-mer peptide of the 2^nd ^extracellular loop of hmAchR M3, which has been suggested as the most important epitope of this receptor protein [31-33] (Figure 3). As a result, we observed a significant difference in IgG reactivity between the PBC patient cohort and healthy controls (Figure 3; bar 1 *vs*. bar 3).

With respect to the elevated frequency of anti-hmAchR M3 IgM-type reactivity in PBC patients (Figure 3; bar 2) we also investigated the control cohort for IgM-type hmAchR M3-specific autoantibodies. However, there was no significant difference in the IgM antibody binding between the PBC patients and the healthy controls (Figure 3; bar 2 *vs*. bar 4).

Differences between the PBC patients being ELISA positive or negative could not be found depending upon the demographic data. However the ELISA positive patients showed a heightened number of patients with elevated IgG (64%) and IgM (91%) levels as compared to the ELISA negative patients (enhanced IgG level: 18%, enhanced IgM level: 69%). With respect to the disease activity we observed that the histologies performed in 6 of the 11 ELISA positive patients, 3 patients showed a disease stage I-II and 3 patients a disease stage III-IV. In 17 out of the 39 patients being ELISA negative histologies were performed from which 12 patients showed a disease stage I-II and 3 patients a disease stage III-IV, while 2 patients were graded as stage II-III.

## Discussion

Autoantibodies to neurotransmitter receptors seem to be involved in the etiopathogenesis of a variety of autoimmune diseases [21-23,26,27]. There are several reports on *in vitro *and *in vivo *studies suggesting a role for autoantibodies to the hmAchR M3 subtype in the etiopathogenesis of the Sjögren's syndrome which often is associated with PBC [26,31,39,40]. This hypothesis is underlined by animal models employing non-obese diabetic (NOD) mice, which are well-known for hypofunction of their salivary glands. Passive transfer of monoclonal antibodies binding specifically to the rat mAchR M3 receptor protein into NOD-scid mice revealed a further diminished secretory function of these glands [41]. In contrast, mice of the Igμ-null-NOD strain (completely lacking functionally active B lymphocytes) do not exhibit any such glandular dysfunction while passive transfer of IgG fraction from patients with M. Sjögren lead to a loss of secretory function [42] and application of F(ab')2 fragments from parental NOD mice diminished it. Concluding from this, autoantibodies to the hmAchR M3 receptor protein are suggested to be responsible for diminished secretion of the salivary gland cells due to an autoantibody-mediated desensitization of the receptor *via *internalization or degradation [43-45].

In our study, we now describe a hitherto unknown co-localization of IgG-type autoantibodies specific to the hmAchR M3 found in the sera of patients with PBC, but not in sera of healthy controls (Figure 1). Of note, not only cell surface membranes were found to be stained by our polyclonal hmAchR M3 antibody; additionally, a diffuse intracellular staining was detectable (Figure 1, lanes A+B, column 2). This effect was not found to be unspecific: employment of three different hmAchR M3-specific antibodies recognizing either (i) the intracellular C-terminus (as used for Figure 1), (ii) the extracellular N-terminal sequence or (iii) the 3^rd ^intracellular domain of the receptor did not change this pattern (data not shown). Therefore, our finding may be in accordance with reports demonstrating internalization of the hmAchR M3 receptor protein [44,45].

Our further results now report a frequency of hmAchR M3-specific autoantibodies in PBC patients in the range of 22-33% (depending upon the detection technique and materials used). Interestingly, these data are comparable with data obtained from the literature for patients with Sjögren's syndrome, in whom autoantibodies were detected by ELISA in a frequency range of 9-40% [32,33,37]. In our study we tested the presence of these antibodies not routinely but only in patients with clinical features of *M. Sjögren*. Thus, identifying only one patient with typical symptoms of *M. Sjögren*, this patient was found to express anti SS-A/SS-B antibodies as detected by ELISA (data not shown). Interestingly, this patient also showed hmAchR M3-specific autoantibodies. Further studies have to show if there is a correlation between anti SS-A/SS-B reactivity and the occurrence of hmAchR M3-specific autoantibodies in PBC patients.

Our observation that autoantibodies to the hmAchR M3 receptor protein could be detected more frequently in Western blotting analysis than in the ELISA system (33% *vs*. 22%) may be due to the fact that in case of Western blotting the whole receptor molecule was used providing a variety of potential epitopes as compared to the peptide used for detection in the ELISA-based assay. Thus, the amino acid sequence of the 2^nd ^extracellular loop as presented in our 25-mer peptide seems to represent not the only epitope, although in the Western blotting analysis a cross-reactivity of autoantibodies to similar muscarinic receptors cannot be excluded completely.

The comparison of the demographic, clinical and biochemical parameters of the patients being anti-M3R positive or negative using the ELISA system, suggests that the anti-M3R antibodies might occur more frequently in patients with an advanced disease. Since, however, in quite a lot of them no biopsies were performed, this observation has to be evaluated in more detail in further studies.

Importantly, hmAchR M3-specific autoantibodies have not been investigated in PBC before. There are, however, two reports on the autoantibody response to nicotinic acetylcholine receptors [46,47] demonstrating the recognition of these proteins by the respective autoantibodies in the majority of the PBC patients as well as patients with *myasthenia gravis*.

Taken together, our studies show again the presence of new specific autoantibodies in PBC patients different to AMA. Since AMA failed to be clearly related to the etiopathogenesis of PBC it is encouraging that several new antibodies have been characterized, which might be associated with disease stage [48] or with new concepts for the etiopathogenesis of PBC [49].

## Conclusions

With respect to the literature and our own data, our concept of the expression of autoantibodies to the muscarinic AchR of the M3 type in patients with PBC could be of importance for the etiopathogenesis of PBC: (i) for the first time, our findings may explain the organ-specificity of the PBC disease since the hmAchR M3 is not expressed on hepatocytes but on cholangiocytes [29]; in addition, also the parasympathetic innervation of the biliary system may point to a role for these autoantibodies. (ii) The hmAchR M3 is also expressed on different glands; in this context it is well-known that PBC patients quite often display an affection of their salivary glands [50]. (iii) Our concept may help to better understand the pathogenesis of PBC if the autoantibodies exert a functional activity in the PBC patients as it was shown for the autoantibodies of the patients with *M. Sjögren *[26,31,39,40]. Therefore, further studies will have to focus on the characterization of the hmAchR M3-specific autoantibodies with respect to an inhibitory or stimulatory effect on the hmAchR.

## Abbreviations

AMA: antimitochondrial antibodies; hmAchR M3: human muscarinic acetylcholine receptor of the M3 type; mAb: monoclonal antibody; OADC: 2-oxoacid dehydrogenase complex; PBC: primary biliary cirrhosis; PDC: pyruvate dehydrogenase complex

## Competing interests

The authors declare that they have no competing interests.

## Authors' contributions

CPB, KB, GMS, and KL performed the experiments. GMS and SW contributed equally to this paper and share senior authorship. GMS, CPB, PAB and SW designed research, analyzed data and wrote the manuscript. MG helped discussing the data. They all read and approved the final manuscript.

## Pre-publication history

The pre-publication history for this paper can be accessed here:

http://www.biomedcentral.com/1471-230X/10/120/prepub

## References

[B1] Gershwin ME Rowley M Davis PA Leung P Coppel R Mackay IR Molecular biology of the 2-oxo-acid dehydrogenase complexes and anti-mitochondrial antibodies Prog Liver Dis 1992 10 47 61 1296237

[B2] Kaplan MM Novosphingobium aromaticivorans: a potential initiator of primary biliary cirrhosis Am J Gastroenterol 2004 99 2147 2149 10.1111/j.1572-0241.2004.41121.x 15554995

[B3] Mackay IR Autoimmunity and primary biliary cirrhosis Baillieres Best Pract Res Clin Gastroenterol 2000 14 519 533 10.1053/bega.2000.0101 10976012

[B4] Sherlock S Scheuer PJ The presentation and diagnosis of 100 patients with primary biliary cirrhosis N Engl J Med 1973 289 674 678 10.1056/NEJM197309272891306 4580473

[B5] Abdulkarim AS Petrovic LM Kim WR Angulo P Lloyd RV Lindor KD Primary biliary cirrhosis: an infectious disease caused by Chlamydia pneumoniae? J Hepatol 2004 40 380 384 10.1016/j.jhep.2003.11.033 15123349

[B6] Dohmen K Shigematsu H Miyamoto Y Yamasaki F Irie K Ishibashi H Atrophic corpus gastritis and Helicobacter pylori infection in primary biliary cirrhosis Dig Dis Sci 2002 47 162 169 10.1023/A:1013292210036 11837719

[B7] Hopf U Moller B Stemerowicz R Lobeck H Rodloff A Freudenberg M Galanos C Huhn D Relation between Escherichia coli R(rough)-forms in gut, lipid A in liver, and primary biliary cirrhosis Lancet 1989 2 1419 1422 10.1016/S0140-6736(89)92034-5 2574361

[B8] Mason AL Xu L Guo L Munoz S Jaspan JB Bryer-Ash M Cao Y Sander DM Shoenfeld Y Ahmed A Van de Water J Gershwin ME Garry RF Detection of retroviral antibodies in primary biliary cirrhosis and other idiopathic biliary disorders Lancet 1989 351 1620 1624 10.1016/S0140-6736(97)10290-2 9620716

[B9] Selmi C Gershwin ME Bacteria and human autoimmunity: the case of primary biliary cirrhosis Curr Opin Rheumatol 2004 16 406 410 10.1097/01.bor.0000130538.76808.c2 15201604

[B10] Xu L Sakalian M Shen Z Loss G Neuberger J Mason A Cloning the human betaretrovirus proviral genome from patients with primary biliary cirrhosis Hepatology 2004 39 151 156 10.1002/hep.20024 14752833

[B11] Berg PA Klein R Immunology of primary biliary cirrhosis Baillieres Clin Gastroenterol 1987 1 675 706 10.1016/0950-3528(87)90053-4 3322437

[B12] Drachman DB How to recognize an antibody-mediated autoimmune disease: criteria Res Publ Assoc Res Nerv Ment Dis 1990 68 183 186 2183310

[B13] Drachman DB Autonomic "myasthenia": the case for an autoimmune pathogenesis J Clin Invest 2003 111 797 799 12639983 10.1172/JCI18180 PMC153777

[B14] Rose NR Bona C Defining criteria for autoimmune diseases (Witebsky's postulates revisited) Immunol Today 1993 14 426 430 10.1016/0167-5699(93)90244-F 8216719

[B15] Witebsky E Rose NR Terplan K Paine JR Egan RW Chronic thyroiditis and autoimmunization J Am Med Assoc 1957 164 1439 1447 13448890 10.1001/jama.1957.02980130015004

[B16] Bjorkland A Totterman TH Is primary biliary cirrhosis an autoimmune disease? Scand J Gastroenterol 1994 204 Suppl 32 39 10.3109/00365529409103623 7824876

[B17] Inamura K Tsuji H Nakamoto Y Suzuki M Kaneko S Transgenic mice aberrantly expressing pyruvate dehydrogenase complex E2 component on biliary epithelial cells do not show primary biliary cirrhosis Clin Exp Immunol 2006 145 93 100 10.1111/j.1365-2249.2006.03090.x 16792678 PMC1941992

[B18] Jones DE Palmer JM Kirby JA De Cruz DJ McCaughan GW Sedgwick JD Yeaman SJ Burt AD Bassendine MF Experimental autoimmune cholangitis: a mouse model of immune-mediated cholangiopathy Liver 2000 20 351 356 10.1034/j.1600-0676.2000.020005351.x 11092252

[B19] Zeniya M Lessons from animal models of primary biliary cirrhosis J Gastroenterol Hepatol 2000 15 342 343 10.1046/j.1440-1746.2000.02170.x 10824874

[B20] Feist E Dorner T Hansen A [Indications and options of new immune modulatory therapies for Sjogren's syndrome] Z Rheumatol 2007 66 679 685 10.1007/s00393-007-0231-z 17999070

[B21] Nguyen VT Ndoye A Grando SA Novel human alpha9 acetylcholine receptor regulating keratinocyte adhesion is targeted by Pemphigus vulgaris autoimmunity Am J Pathol 2000 157 1377 1391 11021840 10.1016/s0002-9440(10)64651-2 PMC1850172

[B22] Jahns R Boivin V Hein L Triebel S Angermann CE Ertl G Lohse MJ Direct evidence for a beta 1-adrenergic receptor-directed autoimmune attack as a cause of idiopathic dilated cardiomyopathy J Clin Invest 2004 113 1419 1429 15146239 10.1172/JCI20149 PMC406525

[B23] Lindstrom JM Nicotinic acetylcholine receptors of muscles and nerves: comparison of their structures, functional roles, and vulnerability to pathology Ann N Y Acad Sci 2003 998 41 52 10.1196/annals.1254.007 14592862

[B24] Mang FW Michieletti P O'Rourke K Cauch-Dudek K Diamant N Bookman A Heathcote J Primary biliary cirrhosis, sicca complex, and dysphagia Dysphagia 1997 12 167 170 10.1007/PL00009532 9190103

[B25] Parikh-Patel A Gold EB Worman H Krivy KE Gershwin ME Risk factors for primary biliary cirrhosis in a cohort of patients from the united states Hepatology 2001 33 16 21 10.1053/jhep.2001.21165 11124815

[B26] Bacman S Sterin-Borda L Camusso JJ Arana R Hubscher O Borda E Circulating antibodies against rat parotid gland M3 muscarinic receptors in primary Sjogren's syndrome Clin Exp Immunol 1996 104 454 459 10.1046/j.1365-2249.1996.42748.x 9099930 PMC2200439

[B27] Gao J Cha S Jonsson R Opalko J Peck AB Detection of anti-type 3 muscarinic acetylcholine receptor autoantibodies in the sera of Sjogren's syndrome patients by use of a transfected cell line assay Arthritis Rheum 2004 50 2615 2621 10.1002/art.20371 15334476

[B28] Alvaro D Alpini G Jezequel AM Bassotti C Francia C Fraioli F Romeo R Marucci L Le Sage G Glaser SS Benedetti A Role and mechanisms of action of acetylcholine in the regulation of rat cholangiocyte secretory functions J Clin Invest 1997 100 1349 1362 10.1172/JCI119655 9294100 PMC508313

[B29] Cassiman D Libbrecht L Sinelli N Desmet V Denef C Roskams T The vagal nerve stimulates activation of the hepatic progenitor cell compartment via muscarinic acetylcholine receptor type 3 Am J Pathol 2002 161 521 530 12163377 10.1016/S0002-9440(10)64208-3 PMC1850744

[B30] Berg CP Stein GM Klein R Pascu M Berg T Kammer W Priemer M Nordheim A Schulze-Osthoff K Gregor M Wesselborg S Berg PA Demonstration of PDC-E1 subunits as major antigens in the complement-fixing fraction M4 and re-evaluation of PDC-E1-specific antibodies in PBC patients Liver Int 2006 26 846 855 10.1111/j.1478-3231.2006.01303.x 16911468

[B31] Cavill D Waterman SA Gordon TP Antibodies raised against the second extracellular loop of the human muscarinic M3 receptor mimic functional autoantibodies in Sjogren's syndrome Scand J Immunol 2004 59 261 266 10.1111/j.0300-9475.2004.01395.x 15030576

[B32] Naito Y Matsumoto I Wakamatsu E Goto D Sugiyama T Matsumura R Ito S Tsutsumi A Sumida T Muscarinic acetylcholine receptor autoantibodies in patients with Sjogren's syndrome Ann Rheum Dis 2005 64 510 511 10.1136/ard.2004.025478 15708912 PMC1755406

[B33] Zigon P Bozic B Cucnik S Rozman B Tomsic M Kveder T Are autoantibodies against a 25-mer synthetic peptide of M3 muscarinic acetylcholine receptor a new diagnostic marker for Sjogren's syndrome? Ann Rheum Dis 2005 64 1247 author reply 1247 16014696 PMC1755593

[B34] Klein R Huizenga JR Gips CH Berg PA Antimitochondrial antibody profiles in patients with primary biliary cirrhosis before orthotopic liver transplantation and titres of antimitochondrial antibody-subtypes after transplantation J Hepatol 1994 20 181 189 10.1016/S0168-8278(05)80056-X 8006398

[B35] Klein R Pointner H Zilly W Glassner-Bittner B Breuer N Garbe W Fintelmann V Kalk JF Müting D Fischer R Tittor W Pausch J Maier KP Berg PA Antimitochondrial antibody profiles in primary biliary cirrhosis distinguish at early stages between a benign and a progressive course: a prospective study on 200 patients followed for 10 years Liver 1997 17 119 128 9249725 10.1111/j.1600-0676.1997.tb00793.x

[B36] Kovacs L Marczinovits I Gyorgy A Toth GK Dorgai L Pal J Molnar J Pokorny G Clinical associations of autoantibodies to human muscarinic acetylcholine receptor 3(213-228) in primary Sjogren's syndrome Rheumatology. Oxford 2005 44 1021 1025 10.1093/rheumatology/keh672 15888503

[B37] Reina S Sterin-Borda L Orman B Borda E Human mAChR antibodies from Sjogren syndrome sera increase cerebral nitric oxide synthase activity and nitric oxide synthase mRNA level Clin Immunol 2004 113 193 202 10.1016/j.clim.2004.08.005 15451477

[B38] Engels IH Stepczynska A Stroh C Lauber K Berg C Schwenzer R Wajant H Jänicke RU Porter AG Belka C Gregor M Schulze-Osthoff K Wesselborg S Caspase-8/FLICE functions as an executioner caspase in anticancer drug-induced apoptosis Oncogene 2000 19 4563 4573 10.1038/sj.onc.1203824 11030145

[B39] Goldblatt F Gordon TP Waterman SA Antibody-mediated gastrointestinal dysmotility in scleroderma Gastroenterology 2002 123 1144 1150 10.1053/gast.2002.36057 12360477

[B40] Waterman SA Gordon TP Rischmueller M Inhibitory effects of muscarinic receptor autoantibodies on parasympathetic neurotransmission in Sjogren's syndrome Arthritis Rheum 2000 43 1647 1654 10.1002/1529-0131(200007)43:7<1647::AID-ANR31>3.0.CO;2-P 10902771

[B41] Nguyen KH Brayer J Cha S Diggs S Yasunari U Hilal G Peck AB Humphreys-Beher MG Evidence for antimuscarinic acetylcholine receptor antibody-mediated secretory dysfunction in nod mice Arthritis Rheum 2000 43 2297 2306 10.1002/1529-0131(200010)43:10<2297::AID-ANR18>3.0.CO;2-X 11037890

[B42] Robinson CP Brayer J Yamachika S Esch TR Peck AB Stewart CA Peen E Jonsson R Humphreys-Beher MG Transfer of human serum IgG to nonobese diabetic Igmu null mice reveals a role for autoantibodies in the loss of secretory function of exocrine tissues in Sjogren's syndrome Proc Natl Acad Sci USA 1998 95 7538 7543 10.1073/pnas.95.13.7538 9636185 PMC22675

[B43] Dawson L Tobin A Smith P Gordon T Antimuscarinic antibodies in Sjogren's syndrome: where are we, and where are we going? Arthritis Rheum 2005 52 2984 2995 10.1002/art.21347 16200578

[B44] Schmidt M Frings M Mono ML Guo Y Oude Weernink PA Evellin S Han L Jakobs KH G Protein-coupled Receptor-induced Sensitization of Phospholipase C Stimulation by Receptor Tyrosine Kinases J Biochem 2000 275 32603 32610 10.1074/jbc.M004784200 10908568

[B45] van Koppen CJ Multiple pathways for the dynamin-regulated internalization of muscarinic acetylcholine receptors Biochemical Society Transactions 2001 29 505 508 10.1042/BST0290505 11498018

[B46] Kyriatsoulis A Manns M Gerken G Lohse AW Maelicke A Wessler I Reske K Meyer zum Büschenfelde KH Immunochemical characterization of anti-acetylcholine receptor antibodies in primary biliary cirrhosis J Hepatol 1988 6 283 290 10.1016/S0168-8278(88)80044-8 3292637

[B47] Sundewall AC Lefvert AK Norberg R Characterization of anti-acetylcholine receptor antibody activity in patients with anti-mitochondrial antibodies Clin Immunol Immunopathol 1987 45 184 195 10.1016/0090-1229(87)90033-X 3665199

[B48] Agmon-Levin N Shapira Y Selmi C Barzilai O Ram M Szyper-Kravitz M Sella S Katz BP Youinou P Renaudineau Y Larida B Invernizzi P Gershwin ME Shoenfeld Y A comprehensive evaluation of serum autoantibodies in primary biliary cirrhosis J Autoimmun 2010 34 55 58 10.1016/j.jaut.2009.08.009 19897339

[B49] Berg CP Thirumalai RK Klein R Gregor M Baseman JB Wesselborg S Lauber K Stein GM Mycoplasma antigen as a possible trigger for the induction of antimitochondrial antibodies in primary biliary cirrhosis Liver Int 2009 29 797 809 10.1111/j.1478-3231.2008.01942.x 19638108

[B50] Palmer JM Doshi M Kirby JA Yeaman SJ Bassendine MF Jones DE Secretory autoantibodies in primary biliary cirrhosis (PBC) Clin Exp Immunol 2000 122 423 428 10.1046/j.1365-2249.2000.01403.x 11122250 PMC1905785

